# Sedative and anesthetic-sparing effects of perioperative full-spectrum cannabis oil in female dogs undergoing unilateral mastectomy and ovariohysterectomy

**DOI:** 10.1007/s11259-026-11367-1

**Published:** 2026-06-25

**Authors:** Ana Gabriela Brito Lima, Vivian Fernanda Barbosa, Renata Regadas, Cinthia Oliveira de Araújo Barreto, Gabriel Menezes Rodrigues, Marcos André Nino Rocha, Herbert Aragão dos Reis, Vitor de Moraes Pina de Carvalho, Laís Pereira Silva, Stella Maria Barrouin-Melo, Alessandra Estrela-Lima

**Affiliations:** 1 Research Center on Mammary Oncology (NPqOM), Veterinary Medicine Hospital (HOSPMEV), Federal University of Bahia, Salvador, 40170-110 Brazil; 2 Department of Anatomy, Pathology and Veterinary Clinics, School of Veterinary Medicine and Animal Science, Federal University of Bahia, Av Milton Santos, 500, Salvador, Bahia 40170-110 Brazil

**Keywords:** Canine mammary tumor, Cannabidiol, Nociception, Perioperative pain, Tetrahydrocannabinol, Veterinary anesthesia

## Abstract

The anesthetic management of female dogs with mammary neoplasia, usually classified as ASA II and undergoing invasive procedures such as mastectomy and ovariohysterectomy, requires effective sedation and anesthetic stability due to the increased anesthetic risk associated with advanced age and underlying disease. In this context, this study aimed to evaluate the sedative effects and reduction in anesthetic requirements of a full-spectrum cannabis oil (FSCO) containing cannabidiol (CBD) and tetrahydrocannabinol (THC) in female dogs undergoing mastectomy and ovariohysterectomy. Twenty dogs were randomly assigned to two groups: group A (*n* = 10), treated with FSCO (0.02 mL/kg PO; 0.2 mg/kg CBD and 0.12 mg/kg THC) twice daily for seven days, plus 0.2 mL/kg (2 mg/kg CBD; 1.2 mg/kg THC) one hour before premedication; and group B (*n* = 10), treated with placebo. Groups A and B had similar ages (9.6; 10.2 years) and weights (7.4; 6.8 kg). Anesthesia was induced with propofol and maintained with sevoflurane. Outcomes included sedation scores, anesthetic requirements, rescue analgesia, responses to instrumentation, and adverse effects. The treated group required less propofol (2.33 vs. 5.98 mg/kg; *p* = 0.001) and lower sevoflurane concentrations from T0 to T4 (*p* < 0.05). Sedation scores were higher at 40 and 60 min (median of 4 vs. 0, and 6.5 vs. 0.5; *p* = 0.015 and *p* = 0.002, respectively). Fewer treated dogs required rescue analgesia (3/10 vs. 6/10; *p* = 0.178). No differences were observed in catheterization, intubation, or adverse effects. Preoperative CBD/THC oil produced sedative effects and reduced anesthetic requirements without clinical complications. These findings support the potential of cannabinoids as safe adjuvants in multimodal anesthesia in veterinary medicine.

## Introduction

Optimizing anesthetic and analgesic protocols in the perioperative period is necessary to maintain physiological stability and improve outcomes in dogs undergoing invasive surgical procedures, such as mastectomy (Giambrone et al. 2025). These canine patients often require prolonged anesthesia, which increases the risk of adverse effects and may extend recovery time (Pinheiro et al. 2023; Giambrone et al. 2025; Horta et al. 2015). However, conventional approaches do not always ensure adequate sedation and anesthetic efficacy, nor do they sufficiently minimize drug exposure and adverse events. This underscores the importance of adjuvant strategies that enhance anesthetic sparing and perioperative stability.

Multimodal anesthesia, which combines various pharmacological approaches, is a widely accepted strategy to achieve superior patient outcomes and reduce the reliance on high doses of individual drugs (Costa and Chaves 2012; Bradbrook and Clark 2018). Within this framework, emerging therapeutic alternatives are continually explored to improve intraoperative stability and postoperative recovery. This is particularly important, as both underdiagnosis and undertreatment of acute pain in the immediate postoperative period, as well as chronic pain in long-term management, continue to be reported issues in canine clinical practice (Rousseau-Blass et al. 2020).

In recent years, phytocannabinoids, such as cannabidiol (CBD) and tetrahydrocannabinol (THC), have garnered increasing interest in veterinary medicine due to their diverse pharmacological properties (Salvo et al. 2023; Shilo-Benjamini etal. 2023). The endocannabinoid system plays a central role in neurophysiological homeostasis and pain modulation in companion animals (Landa et al. 2022; Miranda-Cortés et al. 2023). Full-spectrum *Cannabis sativa* preparations, which contain both CBD and THC, are of particular interest due to their potential synergistic effects (known as the “entourage effect”) that may enhance therapeutic benefits (Blasco-Benito et al. 2018).

THC, acting primarily as a partial agonist at the cannabinoid CB1 receptor in the central nervous system, modulates neurotransmitter release, leading to central effects including sedation (Landa et al. 2022). CBD, while having low direct affinity for CB1 and CB2 receptors, exerts complex modulatory actions via non-cannabinoid targets, such as serotonergic receptors and GABAergic pathways, contributing to anxiolytic and anti-inflammatory properties without marked psychotropic effects (Landa et al. 2016, 2022). These combined mechanisms indicate a potential for cannabinoids to influence perioperative anesthetic requirements.

A reduction in the total dose of anesthetic agents is a highly desirable outcome, especially in oncologic patients who may exhibit increased drug sensitivity and a higher risk of complications (Pinheiro et al. 2023). Cannabinoids have demonstrated an anesthetic-sparing effect, reducing the required doses of drugs such as propofol and sevoflurane in experimental models and clinical settings (Hasckel-Gewehr et al. 2024; Kumar et al. 2010). This effect is very important for optimizing systemic stability, promoting smoother induction, and facilitating a more rapid and comfortable emergence from anesthesia. Moreover, by leveraging these properties, full-spectrum cannabis oil could serve as an adjuvant in multimodal anesthetic protocols, improving the overall safety profile and quality of anesthesia (Hasckel-Gewehr et al. 2024; Kerr and Swanton 2023; Di Salvo et al. 2023).

Clinical research in healthy female dogs undergoing elective ovariohysterectomy indicated that CBD attenuated nociceptive responses and reduced the need for rescue analgesia, supporting its potential role as an adjuvant within multimodal perioperative analgesia (Casas-Alvarado et al. 2024). However, the use of sensitive and validated tools for postoperative evaluation, such as the short form of the Glasgow Composite Measure Pain Scale (GCMPS-SF), is necessary, since they can detect clinically significant differences soon after extubation (Fuertes-Recuero et al. 2024). For this reason, studies on cannabinoid administration in the perioperative setting are required to enrich knowledge on its effectiveness and safety.

Despite these promising findings and the theoretical basis for their application, there is a notable scarcity of controlled studies evaluating the sedative and anesthetic-sparing effects of full-spectrum cannabis oil in the perioperative setting, specifically for female dogs with mammary neoplasia undergoing extensive surgical procedures. Understanding the precise impact of these compounds on anesthetic requirements and immediate perioperative outcomes is essential for their judicious integration into clinical practice.

Therefore, the present study aimed to assess the sedative and anesthetic-sparing effects of a full-spectrum oil containing CBD and THC in female dogs with mammary tumors undergoing unilateral mastectomy combined with ovariohysterectomy. We hypothesized that this formulation would enhance sedation and reduce anesthetic requirements without clinically relevant adverse effects.

## Materials and methods

### Ethical considerations

This study was conducted following the approval of the Ethics Committee on Animal Use (CEUA) of the School of Veterinary Medicine and Zootechny, Federal University of Bahia, under protocol no. 105/2023. All dogs were domiciled, and their owners were informed of the study details through a signed Informed Consent Form.

### Study design and animal selection

**Fig. 1:**
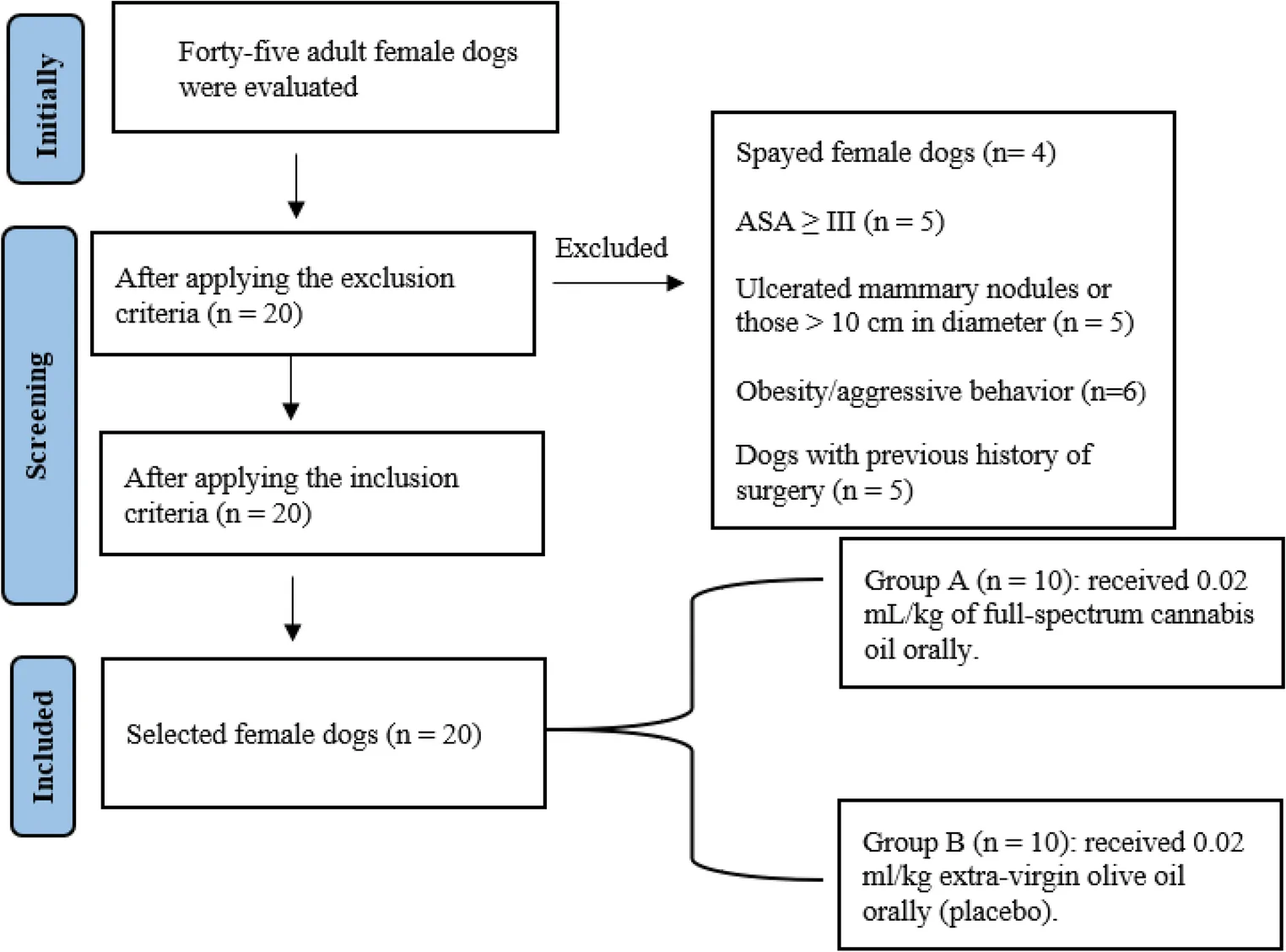
Flowchart for the formation of experimental groups of female dogs pre-treated with cannabis oil (group A) or placebo (group B)

In this prospective, double-blind, placebo-controlled, randomized study, 45 adult female dogs of various breeds diagnosed with mammary tumors and not spayed were initially screened at the Mammary Oncology Research Center of the Federal University of Bahia, Brazil. Of the 45 dogs, 20 were selected to form the study groups, according to the study’s inclusion and exclusion criteria, and were then randomly assigned to two treatment groups (Fig. 1**)**. Randomization was performed using an online platform.

All dogs underwent a complete clinical evaluation, including history, physical examination, and laboratory workup, with complete blood count (CBC), serum biochemistry (urea, creatinine, alkaline phosphatase, total proteins, alanine aminotransferase, and glucose), electrocardiogram, and echocardiogram. Mammary tumors were classified according to the TNM staging system (Cassali et al. 2020; modified from Owen 1980), including tumor size (T), regional lymph node involvement (N), and distant metastasis (M).

The recruited canine patients should meet the following inclusion criteria: (a) Body weight ≤ 12 kg with a body condition of 4 to 5/9, according to Laflamme (2006), to minimize physiological variability and to allow study standardization, since body size may influence anesthetic requirements and pharmacokinetic responses to cannabinoids; (b) Clinically stable, exhibiting only mild systemic disease, or categorized within “Physical Status II” according to the ASA (American Society of Anesthesiologists); (c) Not being spayed; (d) Absence of distant metastases in the oncological staging exams, and (e) Not having previous skin surgery in the ventral thoracic or abdominal regions.

The exclusion criteria were obesity; ASA ≥ III, with ulcerated mammary nodules or those ≥ 10 cm in diameter; being spayed; exhibiting advanced disease, with distant metastases evidenced in oncological staging exams; and having a history of skin surgery in the ventral thoracic or abdominal regions.

The sample calculation was based on a minimum clinically relevant difference of 1.5 mg/kg in the propofol dose between the test group (Group A) and the placebo group (Group B), with an estimated standard deviation of 1.0 mg/kg. A significance level of 5% (alpha) and a statistical power of 80% (beta = 0.20) were adopted, giving an estimated minimum sample size of seven dogs per group. For greater study robustness, after screening, 20 dogs were included and randomly assigned to two groups (*n* = 10 per group).

### Full-spectrum cannabis oil (FSCO) and treatment protocol

The study used a full-spectrum cannabis oil (APEPI, Brazil) containing 10 mg/mL of CBD and 6 mg/mL of THC, characterized by the presence of additional cannabinoids, terpenes, and flavonoids. The composition was verified via high-performance liquid chromatography (HPLC) to ensure precise quantification.

Group A (cannabis) received 0.02 mL/kg of full-spectrum oil orally (equivalent to 0.2 mg/kg CBD and 0.12 mg/kg THC), twice daily (BID), for seven days preoperatively (an acclimatization period carried out at home by the guardian). On the day of surgery, the dogs received a single dose of 0.2 mL/kg (equivalent to 2 mg/kg CBD and 1.2 mg/kg THC) one hour before premedication. This loading dose on the day of surgery was strategically planned to ensure higher plasma levels of cannabinoids during the critical perioperative period, maximizing sedative and anesthetic-sparing effects at the time of induction. Group B (control) received extra-virgin olive oil (placebo) following the same volume and administration schedule as group A.

### Blinding procedures

Vials were prepared and masked by a researcher independent of the trial. Identical color and viscosity characteristics were ensured. Owners were blinded to group allocation during the home administration period. The treatment was administered to the dogs on the day of the surgical procedure by a veterinarian not involved in collecting experimental data.

### Anesthetic and surgical procedures

After an 8-hour food fast and a 2-hour water fast, dogs were premedicated intramuscularly with acepromazine (0.01 mg/kg) and methadone (0.2 mg/kg). Twenty minutes later, a cephalic vein was catheterized and Lactated Ringer’s solution was started at 3 mL/kg/h.

Anesthesia was induced with propofol titrated to effect to allow endotracheal intubation. A total dose of 6.5 mg/kg was prepared initially, and one-quarter of this dose was administered every 20 s until loss of jaw tone and palpebral reflexes permitted intubation (Kastner et al. 2024). Anesthesia was then maintained with sevoflurane in 100% oxygen using a calibrated vaporizer (Tesia 3000; Novitec^®^), with dogs breathing spontaneously. Breathing system selection was based on body weight. Dogs weighing up to 5 kg were managed with a non-rebreathing system to minimize airway resistance and facilitate spontaneous ventilation, whereas dogs weighing more than 5 kg were anesthetized using a circle rebreathing system.

Physiological variables were monitored continuously with a multiparameter monitor (CM120; Dixtal^®^). Recorded variables included esophageal temperature (T°C), heart rate (HR) derived from lead II electrocardiography, mean arterial pressure (MAP) measured oscillometrically using a cuff corresponding to 30–40% of limb circumference placed over the dorsal pedal artery, peripheral oxygen saturation (SpO_2_) measured with the probe positioned on the tongue, respiratory rate (*f*), end-tidal carbon dioxide (EtCO₂), and end-tidal sevoflurane concentration (EtSEVO), both obtained with a gas analyzer.

EtSEVO was adjusted in increments of ± 0.5% to maintain an adequate surgical plane, defined by absence of palpebral reflex, loss of jaw tone, ventromedial eye position, and maintenance of mean arterial pressure between 60 and 90 mmHg. Tumescent local anesthesia was performed using a solution prepared by mixing 2% lidocaine (40 mL) and adrenaline (20 µg/mL) in refrigerated (8 °C) Lactated Ringer’s solution (250 mL) (Vullo et al. 2021). The final solution was administered at 10 mL/kg. All anesthetic procedures were performed by the same anesthetist.

All dogs underwent total unilateral mastectomy and ovariohysterectomy (Fossum 2014), both performed by the same surgical team. One hour after surgery, meloxicam (0.1 mg/kg, SC) was administered.

### Clinical assessments

**Fig. 2:**
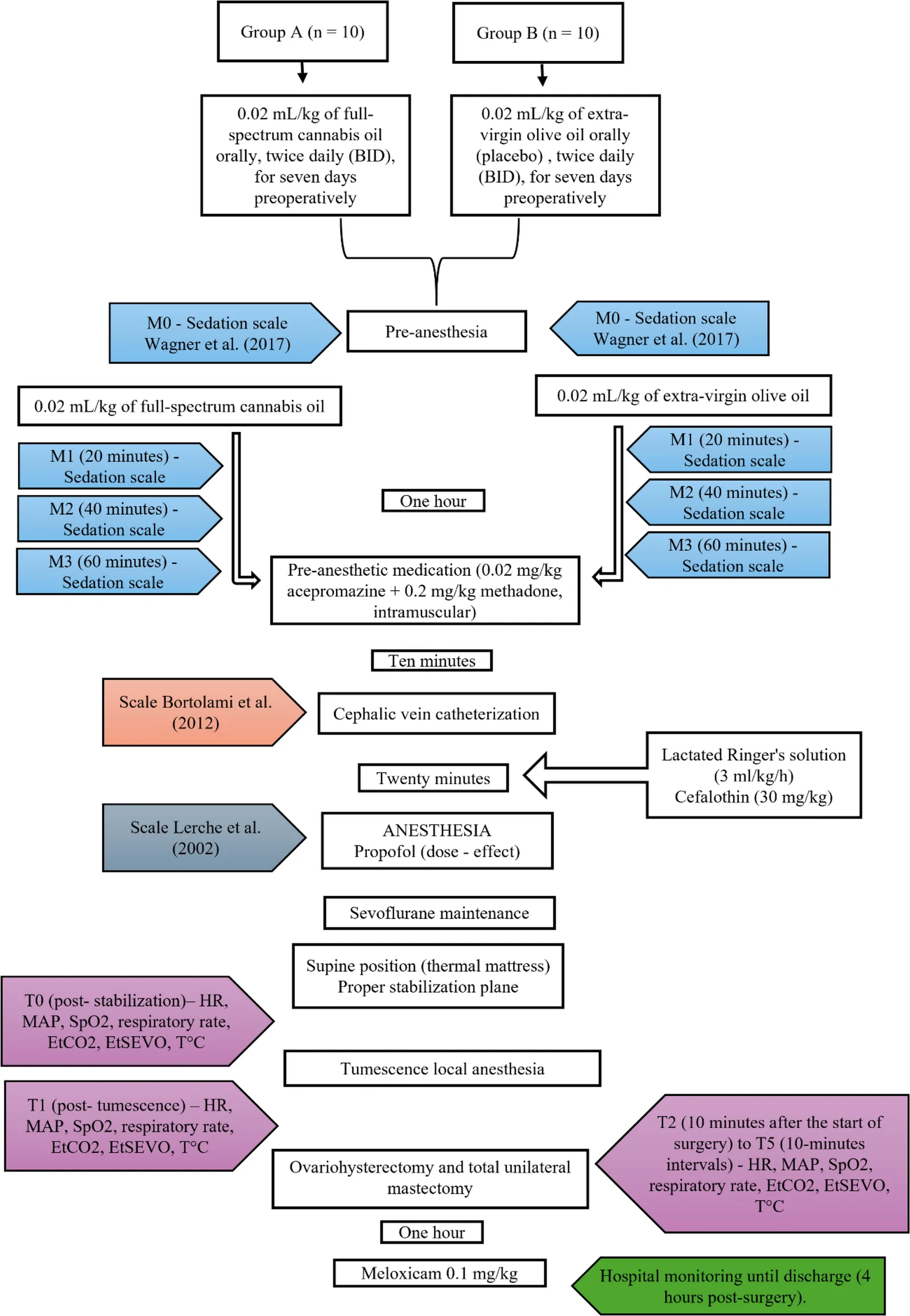
Flowchart of the experimental design of the study. Application of scales and evaluation of physiological parameters - HR: Heart rate; MAP: mean arterial pressure, SpO2: Oxygen saturation, EtCO2: end-tidal CO2, EtSEVO: End-tidal sevoflurane concentration, and T°C: temperature in degrees Celsius

*Sedation scores:* evaluated at 0 (baseline), 20, 40, and 60 min after oil/placebo (Fig. 2) administration using a validated composite scale ranging from 0 to 12 points, in which higher scores indicate deeper sedation based on behavioral and physical parameters such as posture, response to handling, muscle relaxation, and alertness (Wagner et al. 2017). Each parameter is scored on a 0–4 scale, yielding a total score ranging from 0 (no sedation) to 12 (maximum sedation).

*Instrumentation scores*: response to venous catheterization (0–3) (Bortolami et al. 2013) and quality of intubation (0–3) (Lerche et al. 2002). The venous catheterization response scale described by Bortolami et al. (2013) was interpreted as follows: 0 = no reaction, with the dog remaining immobile in sternal recumbency; 1 = slight limb movement and mild muscle tension, requiring minimal restraint; 2 = limb withdrawal and escape attempts, requiring moderate restraint; 3 = marked escape behavior, vocalization and/or aggression, requiring strong restraint and multiple catheterization attempts. Endotracheal intubation conditions followed the scale proposed by Lerche et al. (2002): 0 = easy intubation on the first attempt, without coughing or laryngospasm; 1 = intubation on the first attempt with coughing or slight movement; 2 = intubation requiring more than one attempt, with or without movement; 3 = failed intubation due to jaw rigidity, laryngospasm, or excessive movement.

*Physiological parameters and maintenance anesthetic requirement: *HR, MAP, SpO_2_, *f*, T°C, EtCO_2_ and EtSEVO were recorded after stabilization (T0), before incision (T1), and every 10 min during surgery (T2–T5) (Fig. 2).

*Rescue analgesia:* Intraoperative rescue analgesia with fentanyl (2.5 µg/kg IV) was administered when HR or MAP increased by 20% or more from the pre-incisional baseline value, despite EtSEVO adjustment to maintain an adequate anesthetic plane. Both the number of rescue analgesia interventions per animal and the number of dogs requiring rescue analgesia were recorded.

*Adverse events:* guardian-reported behavioral changes during home administration. Hospital monitoring (4 h post-surgery) included bradycardia, hypotension, vomiting, or diarrhea until discharge, as reported by Amissah et al. (2022) and McGrath et al. (2018).

### Statistical analysis

Data normality was assessed using the Kolmogorov-Smirnov test. Categorical variables were compared using Fisher’s exact test. For continuous variables, comparisons between groups were performed using the independent Student’s t-test (parametric) or the Mann-Whitney U test (non-parametric). Comparisons across time points were conducted using one-way ANOVA followed by Tukey’s post-hoc test for parametric data. In contrast, the Kruskal-Wallis test followed by Dunn’s post hoc test was used for the nonparametric longitudinal analysis. Statistical significance was set at *p* < 0.05. Analyses were performed using GraphPad Prism 8.0 (GraphPad Software, San Diego, CA, USA) and IBM SPSS Statistics 26.0 (IBM Corp., Armonk, NY, USA).

## Results

### Demographic data and surgical duration

No significant differences were observed between groups regarding age (*p* = 0.57), body weight (*p* = 0.369), surgery duration (*p* = 0.336), or anesthesia duration (*p* = 0.511). The mean age was 9.6 ± 1.9 years for group A and 10.2 ± 2.6 years for group B. The mean weight was 7.4 ± 0.8 kg for group A and 6.4 ± 0.8 kg for group B. The mean surgery time was 44.1 ± 1.7 min in group A and 42.6 ± 1.4 min in group B, and the anesthesia duration was 58.7 ± 2.6 and 56.5 ± 3.1 min, respectively (Table 1).

**Table 1:** Demographic characteristics and procedural times in female dogs pre-treated with full-spectrum cannabis oil (group A) or placebo (group B)

Parameters	Group A (*n* = 10)	Group B (*n* = 10)	*p*-value
Weight (kg)	7.4 ± 0.8	6.4 ± 0.8	0.369
Age (years) Surgery duration (min)	9.6 ± 1.9 44.1 ± 1.7	10.2 ± 2.6 42.6 ± 1.4	0.57 0.336
Anesthesia duration (min)	58.7 ± 2.6	56.5 ± 3.1	0.511
Breed n (%)			0.268
Mixed breed	4 (40%)	3 (30%)	
Poodle	2 (20%)	3 (30%)	
Yorkshire / Pinscher	1 (10%) / 0	2 (20%) / 2 (20%)	
Others	3 (30%)	0	

Data expressed as mean ± standard deviation (SD) or frequency (%)

### Sedation and instrumentation scores

Dogs treated with full-spectrum oil (group A) exhibited significantly higher sedation scores at 40 min (M2; *p* = 0.015) and 60 min (M3; *p* = 0.002) compared to the placebo group (Table 2). No significant differences were found in behavioral responses to venous catheterization (*p* = 0.453) or orotracheal intubation (*p* = 0.653) between the groups.

**Table 2:** Sedation scores (0–12) at baseline (M0) and intervals after treatment (M1–M3)

Group	M0	M1	M2	M3
A	0 (0–3)ᵃ	1 (0–5)ᵃ	4 (0–7)ᴬᵇ	6.5 (1–8)ᴬᵇ
B	0 (0–2)	0 (0–2)	0 (0–2)ᴮ	0.5 (0–3)ᴮ

Values expressed as median (min–max). Different uppercase letters in columns indicate a statistical difference between groups (*p* < 0.05). Different lowercase letters in rows indicate a difference between time points (*p* < 0.05)

### Anesthetic requirements

**Fig. 3:**
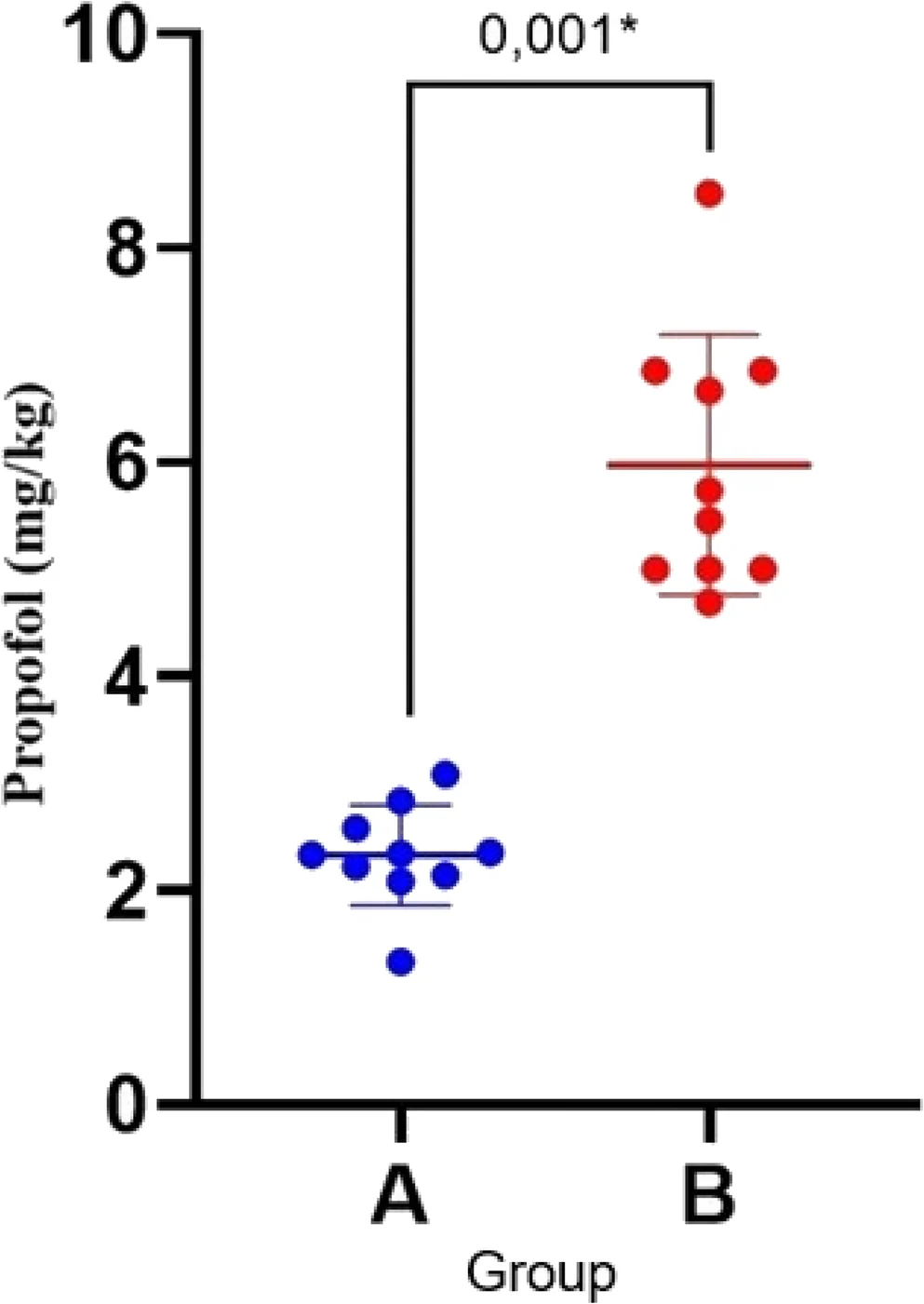
Individual requirements for propofol for anesthetic induction and its median in female dogs previously treated with full-spectrum cannabis oil (**A**) or placebo (**B**)

A major finding was the significant reduction in propofol requirements for induction in group A (2.33 mg/kg) compared to group B (5.98 mg/kg; *p* = 0.001), representing a 61% anesthetic-sparing effect **(**Fig. 3).

EtSEVO varied significantly between the groups. The treated group (Group A) exhibited a lower sevoflurane requirement at time points T0 (*p* = 0.048), T1 (*p* = 0.005), T2 (*p* = 0.007), T3 (*p* = 0.001), and T4 (*p* < 0.001). In the control group (Group B), an increase in the baseline (T0) halogenated anesthetic requirement was observed from 20 min after the onset of the surgical procedure (T2; *p* = 0.006), as well as at T3 (*p* = 0.006) and T4 (*p* = 0.004), while at T5 there was no significant difference (Table 3).

**Table 3:** End-tidal sevoflurane concentration (EtSEVO, %) during the intraoperative period

Group	T0	T1	T2	T3	T4	T5
A	1.1 ± 0.6ᴬ	1.0 ± 0.6ᴬ	1.6 ± 0.7ᴬ	1.5 ± 0.6ᴬ	1.4 ± 0.6ᴬ	1.7 ± 0.6
B	1.6 ± 0.5ᴮᵃ	1.8 ± 0.5ᴮ	2.5 ± 0.6ᴮᵇ	2.5 ± 0.4ᴮᵇ	2.5 ± 0.4ᴮᵇ	2.2 ± 0.5

Mean ± SD. Different uppercase letters (columns) indicate differences between groups. Different lowercase letters (rows) indicate differences between time points

### Physiological parameters

The physiological parameters are shown in Table 4. Mean heart rates were significantly lower for group A compared to group B at T0 (*p* = 0.001), T1 (*p* = 0.028), T2 (*p* = 0.009), T3 (*p* = 0.002), and T5 (*p* = 0.043). No significant variations were detected between treatments or over time for respiratory rate (*f*), SpO_2_, EtCO_2_, or mean arterial pressure (MAP).

**Table 4:** Physiological variables during the intraoperative period

Group		T0		T1	T2	T3	T4	T5
HR	A	72 ± 17^A^	85 ± 24^A^		70 ± 21^A^	74 ± 21^A^	83 ± 21	83 ± 21^A^
	B	119 ± 35^B^	110 ± 22^B^		97 ± 20^B^	104 ± 15^B^	100 ± 15	105 ± 16^B^
*f*	A	16.2 ± 7.7	15.6 ± 9.0		17.0 ± 9.5	16.8 ± 8.7	16.8 ± 6.1	12.1 ± 6.3
	B	21.4 ± 13.2	15.9 ± 8.6		15.0 ± 6.4	13.8 ± 6.0	16.1 ± 7.4	15.9 ± 6.7
SpO_2_	A	99.4 ± 0.7	99.7 ± 0.7		98.7 ± 2.1	99.3 ± 1.6	98.9 ± 1.9	99.1 ± 1.9
	B	99.1 ± 1.7	99.4 ± 0.9		99.2 ± 1.0	99.7 ± 0.5	99.7 ± 0.5	99.2 ± 0.9
EtCO_2_	A	35.3 ± 8.9	35.9 ± 4.7		38.5 ± 7.3	35.4 ± 7.0	35.0 ± 7.8	37.6 ± 6.6
	B	35.5 ± 4.4	36.0 ± 4.2		38.6 ± 5.1	37.2 ± 3.2	38.9 ± 5.0	38.4 ± 4.2
MAP	A	81.6 ± 21.5	71.3 ± 12.1		77.4 ± 16.6	74.9 ± 17.0	76.8 ± 11.3	84.3 ± 12.6
	B	86.4 ± 14.1	76.0 ± 12.5		81.3 ± 28.9	79.2 ± 8.9	79.9 ± 11.9	82.0 ± 9.6

Mean ± SD. Different uppercase letters (columns) indicate difference between groups

### Analgesic rescues and adverse events

There was no difference in the number of dogs requiring rescue analgesia between the treated (3/10) and placebo (6/10) groups (*p* = 0.178). In all of these animals, a single rescue analgesia administration was sufficient. No adverse events were reported during at-home treatment administration or during the immediate post-anesthetic period (approximately 4 h).

## Discussion

The results of this study demonstrate that preoperative administration of full-spectrum cannabis oil (containing CBD and THC) promoted significant sedative and anesthetic-sparing effects in female dogs undergoing mastectomy and ovariohysterectomy. As previously raised, full-spectrum Cannabis sativa oil, composed of different phytocannabinoids in synergistic association, enhanced therapeutic effects while reducing the undesirable side effects of drugs, a phenomenon known as the “entourage effect”, in mice (Blasco-Benito et al. 2018).

The 61% reduction in propofol requirements and the significantly lower sevoflurane consumption observed in the dogs of our study highlight the potential of cannabinoids as adjuvants in multimodal anesthesia. This marked reduction in drug consumption was attributed to the effect of cannabinoids as anesthetic-sparing agents within multimodal protocols. This effect may be explained by the central action of THC through CB1 receptors, which are widely expressed in the central nervous system, enhancing sedative and analgesic effects via modulation of neurotransmitter release (Landa et al. 2022).

We observed that one placebo-treated dog required a higher propofol dose than anticipated. Because induction was titrated to predefined intubation criteria, this finding most likely reflects recognized interindividual variability in anesthetic sensitivity in dogs rather than a protocol-related effect. This point has been raised before by other authors (Sahinovic et al. 2018). However, in our study, the statistical approach minimized the influence of this isolated value on overall group comparisons.

Indeed, CBD exerts modulatory actions across multiple non-cannabinoid pathways, including serotonergic systems, TRPV1 (Transient Receptor Potential Vanilloid 1), adenosine signaling, and GABAergic mechanisms, which collectively contribute to reduced nociception, improve overall analgesic control, and lower anesthetic requirements (Landa et al. 2016, 2022). Clinically, such anesthetic-sparing effects may be particularly relevant in oncologic patients, in whom minimizing anesthetic exposure is often desirable to optimize systemic stability and postoperative recovery.

The significantly lower sevoflurane requirement in dogs treated with full-spectrum *Cannabis* oil persisted throughout nearly the entire intraoperative period, further reinforcing the anesthetic-sparing potential of cannabinoids with inhalant agents. This reduction may have resulted from an adjunctive effect of *Cannabis* oil on anesthetic stability and intraoperative analgesic modulation. This is consistent with experimental findings in which cannabinoid agonists prolonged isoflurane anesthesia in murine models (Schuster et al. 2002). Nevertheless, given the complex and bidirectional interactions between CBD and THC within the endocannabinoid system, it is likely that overlapping pharmacodynamic mechanisms, similar to those that contribute to the reduced propofol requirement, also underlie the anesthetic-sparing effect of sevoflurane.

The doses and treatment duration used in this study were based on established safety profiles (Bartner et al. 2018; Gamble et al. 2018). It is worth noting that a preoperative loading dose was strategically used to achieve higher plasma levels during induction, aiming to enhance the sedative and propofol-sparing effects. The oral route was chosen due to its superior bioavailability and systemic absorption in dogs. Our findings align with previous studies reporting that ECS modulation reduced anesthetic doses in rodents and dogs (Kumar et al. 2010; Hasckel-Gewehr et al. 2024).

The mechanism behind this sparing effect likely involves both pharmacodynamic synergism and pharmacokinetic interactions. CBD acts as a negative allosteric modulator of CB1 receptors and inhibits the degradation of anandamide (AEA) by FAAH (Laprairie et al. 2015). Furthermore, cannabinoids may inhibit cytochrome P450 enzymes (CYP2C9 and CYP3A4), which are responsible for propofol metabolism, potentially increasing its plasma half-life and hypnotic effect (Baker et al. 2025).

Interestingly, while human studies often report that chronic cannabis use *increases* anesthetic requirements due to receptor desensitization (Bornemann-Cimenti et al. 2025), our 7-day preoperative protocol in dogs suggested a beneficial “sensitization” or adaptive modulation of the ECS. This highlights the importance of species-specific protocols and the “entourage effect” of full-spectrum oils, which may mitigate the tolerance typically seen with isolated compounds (Blasco-Benito et al. 2018).

All dogs received multimodal intraoperative analgesia consisting of opioid premedication combined with tumescence local anesthesia, a technique known to provide effective regional analgesia for mastectomy procedures (Kerr and Swanton 2023; Giambrone et al. 2025). This robust baseline protocol likely contributed to the low requirement for rescue analgesia while still allowing detection of additional anesthetic-sparing effects in treated dogs.

The lack of superior analgesic effects observed in the cannabinoid-treated group may be explained by several factors, including the multimodal analgesic protocol used in the present study, the distinct pathophysiological characteristics of acute surgical nociception compared with chronic inflammatory pain models where cannabinoids have been more investigated, and the relatively small sample size, which may have limited the statistical power to detect differences between groups.

The increased sedation observed at 40- and 60-minute post-administration, without adverse hemodynamic effects, might have resulted from the anxiolytic properties of CBD exerted through 5-HT1A receptor interaction and HPA axis regulation. As raised previously, these mechanisms collectively support an anxiolytic and sedative effect (Landa et al. 2022; Hunt et al. 2023; Opyd 2025). Additionally, other authors discuss that cannabinoid signaling may reduce neurotransmitter release through Gi/o protein–coupled pathways, leading to inhibition of voltage-dependent Ca²⁺ channels and modulation of K⁺ channels, thereby contributing to sedation without promoting hemodynamic instability (Miranda-Cortés et al. 2023). This is particularly valuable for oncological patients, who often present with increased sensitivity and stress.

The relative intraoperative bradycardia observed in the treated group reinforces the sedative effect of cannabinoids and rules out deleterious cardiovascular depression or hypotension, given the stability and similarity of MAP between the groups. This observation confirms the cardiovascular safety of the protocol under evaluation. Cannabinoids may influence cardiovascular function through both central and peripheral mechanisms; an activation of CB1 receptors in vascular and myocardial tissues is associated with hypotensive and cardiodepressant responses in vivo, indicating that endocannabinoid signaling modulates autonomic cardiovascular control and vascular tone (Pacher et al. 2005). In addition, reductions in sympathetic outflow and peripheral vasodilation may contribute to decreases in blood pressure and heart rate (Cunha et al. 2011). In the present study, the relative bradycardia observed in the treated group was not accompanied by clinically relevant hypotension or significant changes in mean arterial pressure. This supports the interpretation that these cardiovascular effects reflect autonomic modulation associated with sedation rather than an adverse hemodynamic depression.

The implications of prior cannabinoid receptor sensitization in the present study are clinically significant. We hypothesize that pretreatment not only facilitated more effective modulation of perioperative pain and anxiety but also influenced the pharmacological interaction with conventional anesthetic agents. The observed anesthetic-sparing effect may have resulted from optimized modulation of the endocannabinoid system, which, instead of leading to tolerance, may have increased responsiveness to the sedative and analgesic actions of cannabinoids. In turn, these effects may have complemented standard anesthetics and contributed to the reduction in anesthetic requirements identified in this study.

Several mechanisms may underlie this phenomenon, including the possible upregulation of specific receptor pathways or alterations in the synthesis and degradation of endogenous cannabinoids, resulting in a neurochemical environment more permissive to analgesic and anesthetic modulation (McPartland et al. 2014). However, these interpretations remain speculative, and further studies are needed to clarify the extent to which prior exposure to cannabinoids modulates anesthetic sensitivity in surgical patients.

While these results are promising, they should be interpreted with caution, given the limited sample size and focus on the immediate perioperative period. Furthermore, other limitations include the lack of formal postoperative pain assessment using validated pain scales and the absence of objective nociception-monitoring tools (e.g., PTA index). Postoperative biochemical monitoring (e.g., liver enzymes) also was not performed and should be investigated in further studies on the adjuvant effect of cannabinoids on anesthesia of dogs with invasive surgeries. Because only dogs weighing ≤ 12 kg were enrolled to enhance anesthetic standardization, the extrapolation of these results to larger dogs should be made with caution and warrants further investigation. Future longitudinal studies with larger cohorts are warranted to explore the long-term benefits of full-spectrum cannabis oil on postoperative recovery and pain management in veterinary medicine.

## Conclusion

The findings of this study indicate that preoperative administration of full-spectrum *Cannabis sativa* oil containing CBD and THC exerts significant sedative and anesthetic-sparing effects in female dogs with mammary neoplasia undergoing mastectomy and ovariohysterectomy. The use of this phytocannabinoid extract significantly reduced the required doses of both propofol for induction and sevoflurane for maintenance, supporting its potential as an adjuvant in multimodal anesthetic protocols. Furthermore, the therapy was not associated with clinical adverse effects or compromised anesthetic safety, suggesting a favorable safety profile for perioperative use in oncological patients.

## Data Availability

The datasets generated during and/or analyzed during the current study are available from the corresponding author on reasonable request.
